# Using Novel Statistical Techniques to Accurately Determine the Predictive Dose Range in a Study of Overall Survival after Definitive Radiotherapy for Stage III Non-Small Cell Lung Cancer in Association with Heart Dose

**DOI:** 10.4236/jct.2021.129044

**Published:** 2021-09-29

**Authors:** Joshua R. Niska, Jiuyun Hu, Jing Li, Michael G. Herman, Cameron S. Thorpe, Steven E. Schild, Mirek Fatyga

**Affiliations:** 1 Department of Radiation Oncology, Banner MD Anderson Cancer Center, Phoenix, Arizona, USA; 2 School of Computing, Informatics, and Decision Systems Engineering, Tempe, Arizona, USA; 3 School of Industrial and Systems Engineering, Georgia Institute of Technology, Atlanta, Georgia, USA; 4 Department of Radiation Oncology, Mayo Clinic, Rochester, Minnesota, USA; 5 Department of Radiation Oncology, Mayo Clinic, Phoenix, Arizona, USA

**Keywords:** Lung Cancer, Cardiac Toxicity, Lung Radiation Therapy, Non-Small Cell Lung Cancer, Radiation Toxicity

## Abstract

**Purpose:**

Recent studies of radiotherapy (RT) for stage III non-small-cell lung cancer (NSCLC) have associated high dose to the heart with cardiac toxicity and decreased overall survival (OS). We used advanced statistical techniques to account for correlations between dosimetric variables and more accurately determine the range of heart doses which are associated with reduced OS in patients receiving RT for stage III NSCLC.

**Methods:**

From 2006 to 2013, 119 patients with stage III NSCLC received definitive RT at our institution. OS data was obtained from institutional tumor registry. We used multivariate Cox model to determine patient specific covariates predictive for reduced overall survival. We examined age, prescription dose, mean lung dose, lung V20, RT technique, stage, chemotherapy, tumor laterality, tumor volume, and tumor site as candidate covariates. We subsequently used novel statistical techniques within multivariate Cox model to systematically search the whole heart dose-volume histogram (DVH) for dose parameters associated with OS.

**Results:**

Patients were followed until death or 2.5 to 81.2 months (median 30.4 months) in those alive at last follow up. On multivariate analysis of whole heart DVH, the dose of 51 Gy was identified as a threshold dose above which the dose volume relationship becomes predictive for OS. We identified V55Gy (percentage of the whole heart volume receiving at least 55 Gy) as the best single DVH index which can be used to set treatment optimization constraints (Hazard Ratio = 1.044 per 1% increase in heart volume exposed to at least 55 Gy, P = 0.03). Additional characteristics correlated with OS on multivariate analysis were age, stage (IIIA/IIIB), and administration of chemotherapy.

**Conclusion:**

Doses above 51 Gy, applied to small volumes of the heart, are associated with worse OS in stage III NSCLC patients treated with definitive RT. Higher stage, older age and lack of chemotherapy were also associated with reduced OS.

## 1. Introduction

Late cardiac effects years to decades after thoracic radiotherapy (RT) have been well-described [1]. However, recent prospective studies in lung cancer have identified radiation-related cardiac events occurring on an earlier timeframe of months to years. Heart dose volume histogram (DVH) variables associated with cardiac toxicity in these prospective studies include: mean heart dose (MHD) [2] [3] [4], heart V5 (volume of the heart receiving at least 5 Gy) [2] [3] [4] [5], heart V30 [2] [3], heart V35 [6], heart V55 [4], heart V60 [5], mean left ventricle dose [3] [5], left ventricle V5 and V30 [3] [5], mean left atrium dose [5], left atrium V30 [5], and right atrium V60 [5].

Heart dose has not only been associated with cardiac events [2] [3] [4] [5] [6] in prospectively evaluated patients but also overall survival (OS) [7] [8] [9]. In contrast to expectations, the Radiation Therapy Oncology Group (RTOG) 0617 trial demonstrated decreased OS in patients randomized to higher dose radiotherapy for locally advanced non-small-cell lung cancer (NSCLC) [7]. Subsequent analysis of RTOG 0617 associated increased heart V40 [8] and heart V50 [10] with decreased OS.

Prior published studies determined a wide range of associations between dosimetric variables and OS which made their consistent clinical application difficult and even raised doubts as to the veracity of the findings [11]. The inconsistencies among studies are most likely attributable to correlations among Dose Volume Histogram (DVH) variables which were not accounted for in a conventional statistical analysis. To address these shortcomings, we used advanced statistical techniques to systematically search for heart DVH variables which were most predictive for the OS in a cohort of 119 patients, treated for stage III NSCLC with definitive RT at Mayo Clinic Arizona (MCA). We selected well established statistical techniques which are applicable to highly correlated covariates but also enhanced them with novel constraints which reflect radiobiological knowledge specific to RT. These additional constraints improve generalizability of the model and make the results easier to interpret.

The increasing use of intensity modulated RT (IMRT) and proton beam therapy (PBT) may allow for more precise cardiac sparing radiation plans, provided that evidence based treatment planning constraints on heart dosimetry can be reliably established.

## 2. Methods and Materials

### 2.1. Patient Characteristics

From 2006 to 2013 at Mayo Clinic Arizona, 119 stage III NSCLC patients were treated with definitive RT. RT was delivered using involved-field technique and either 3-dimensional conformal RT (3D-CRT) or IMRT. Patient characteristics are shown in Table 1.

### 2.2. Treatment Planning Heart Constraints

Typical dose constraints on the whole heart structure during treatment planning were: Maximum dose < 62 Gy, Mean dose < 26 Gy, *V*_30Gy_ < 46% ; *V*_40Gy_ < 33%.

### 2.3. Heart Dose Extraction

The whole heart was contoured for each patient on the radiation planning computed tomography (CT) image using Eclipse treatment planning system (Varian, Inc). Dosimetric information was extracted from Eclipse using the Eclipse Application Programmer Interface (ESAPI) software and reprocessed for statistical analysis using proprietary institutional software. The typical planning image was acquired with axial spacing of 2 mm and 1 - 2 mm voxels in the anterior-posterior and medial-lateral dimensions. Dose extraction was performed using a rectangular grid with the dimensions of the planning image.

### 2.4. Dosimetric Analysis

The dose to the heart was quantified using dosimetric index *V_D_* which was defined as the percentage of the volume of the heart (or heart segment) receiving dose ≥ *D* in Gy. For each dose-volume histogram (DVH), a range of *V_D_* indices was extracted, with dose D varying in 1 Gy steps between 5 Gy and 60 Gy. The dose was not converted to biologically equivalent dose as all treatments were conventionally fractionated photons at 1.8 - 2.0 Gy per fraction.

### 2.5. Statistical Analysis

Possible association between heart dosimetry and OS is most commonly investigated using the multivariate Cox model with heart dosimetry represented by preselected heart DVH variables (e.g., a *V_D_* which is the percentage of heart volume receiving dose *D*, or greater). The drawback of this approach is that p-values associated with DVH variables need to be scaled by False Discovery Rate (FDR) correction. Since the dosimetric variables are highly correlated, the FDR correction can be overly strict and therefore may find none of the dosimetric variables being significant, particularly in studies with limited patient numbers. Also, this approach cannot assess the joint effect of dosimetric variables on OS. To address these limitations a multivariate Cox model can be adopted in which heart dosimetry is represented by the linear combination of DVH features, *i.e.*,:

$$h\left(t\right)=h_{0}\left(t\right)\exp\left(\beta_{0}+\beta_{1}X_{1}+\cdots +\beta_{p}X_{p}\right),$$

which links the hazard function *h*(*t*) with dosimetric variables *X*_1_,⋯,*X_p_*. For example *X*_1_,⋯,*X_p_* can be *V*_5GY_,*V*_6Gy_,⋯,*V*_59Gy_,*V*_60Gy_. The conventional multivariate Cox model may suffer from multicollinearity due to the high correlation between the dosimetric variables. Another challenge is the curse of dimensionality as the number of dosimetric variables included in the multivariate model is typically quite large compared with the limited sample size. To address these issues, we took advantage of modern developments in statistical analysis by adding constraints on the coefficient estimates, which are known as the “variable selection techniques” [12]. The well-known Lasso model [13] adds an L1 penalty on the coefficient estimates, *i.e.*, $\sum_{i-1}^{p}\left|\beta_{i}\right|<s$, which has the effect of suppressing the small-effect coefficients to be zero and thus selecting the subset of important dosimetric variables simultaneously. To further account for the high correlation among the dosimetric variables, the fused Lasso model [14] includes an additional L1 penalty on dosimetric variables with adjacent dose levels *i.e.*, $\sum_{i-1}^{p}\left|\beta_{i}-\beta_{i-1}\right|<\gamma$. The upper bounds in these constraints, *s* and *γ*, are selected using a grid search to optimize a commonly used model selection criterion. Based on the work of Dai and Breheny [15] we use leaveoneout cross validation of linear predictors during the grid search to find parameters *s* and *γ* associated with the lowest cross validation error (Supplement S2.2).

In this paper, we adopted the fused Lasso as our base formulation but added two additional constraints inspired by biomedical domain knowledge. The resulting model is called knowledge-constrained Lasso (KC-Lasso). The two constraints are non-negativity and monotonicity of coefficients for dosimetric variables. The non-negativity means that we require *β_i_* ≥ 0, *i* = 1,⋯, *p*. This constraint reflects a biologically motivated hypothesis that increasing *V_D_* poses either a higher hazard risk or no significant risk but cannot lower the risk. The monotonicity constraint specifies that *β*_1_ ≤ *β*_2_ ≤ ⋯ ≤ *β_p_* where *β*_1_ to *β_p_* are coefficients corresponding to *V_D_*’s with *D* from the lowest to the highest dose levels and is motivated by radiobiology. If the same volume is irradiated to a higher dose, the hazard ratio associated with the irradiation cannot decrease since higher doses are always associated with lower cell survival fractions. Lower cell survival fraction may keep clinical toxicity the same or make it worse, but it cannot make it better (Supplement S2.1). Integrating the non-negativity and monotonicity constraints into the fused Lasso formulation, the resulting KC-Lasso model can be estimated by the “penalized” function in the R package.

The analysis was performed in two distinct steps: In the first step we selected patient-specific covariates which were predictive for the OS, using Cox model with Akaike Information Criterion (AIC). Following covariates were considered: stage, chemotherapy, age, prescription dose, mean lung dose, lung V20, tumor site, and laterality. Tumor volume was not included as a candidate covariate because of strong correlation between volume and stage (Supplement S2.5). In the second step we retained only predictive patient specific covariates and additionally included dosimetric covariates, without penalizing patient specific covariates.

We applied the KC-Lasso model with two different resolutions, one with 1 Gy spacing between indices, *i.e.*, 5 Gy, 6 Gy, ⋯, 59 Gy, 60 Gy included in the model, which we called the “dense fit”; and the other with 5 Gy spacing, *i.e.*, 5 Gy, 10 Gy, ⋯, 55 Gy, 60 Gy, which we called the “sparse fit”. The selected patient-specific covariates were included in each model. The specific purpose of varying the dose step was to test the self-consistency of the approach as the step size selection is arbitrary and the final evaluation of the Hazard Ratios should not depend strongly on the step size selection. Greater resolution provides a better estimate of the effective dose threshold and the final Hazard Ratio, at a cost of greater complexity of the model in its final applications.

The KC-Lasso model can identify the dose threshold (or thresholds) beyond which the dose volume variables, *V*_*D*≥D*_ become predictive for OS. However, the model itself cannot be used directly in commercially available optimization packages which set constraints on individual indices only. For this reason we also fit a family of multivariate Cox models, each with only one *V_D_* index representing heart dosimetry, to obtain a (slightly less precise) model which is directly usable as a dose constraint in commercially available treatment planning systems. Since KC-Lasso identifies the range of doses which are predictive for OS, we did not scale p-values in the simplified approach by the FDR correction if they fell within the range indicated by KC-Lasso. In clinical applications the single index constraint can be used to set the optimization constraint based on limiting the Hazard Ratio, while the full formulation of KC-Lasso can be used for final evaluation of the Hazard Ratio in the plan.

KC-Lasso is not the only statistical methodology that could be applied to DVH analysis. The alternatives could include Lasso and Fused Lasso methods without knowledge constraints or entirely different statistical approaches which account for correlations among indices. To explore potential alternatives, we applied Lasso, Fused Lasso, and Elastic Net [16] models to our data set and compared the results to KC-Lasso.

## 3. Results

Median follow-up for all patients was 18 months (range 1.1 to 81.2 months). At last follow-up, 47 patients (39.5%) were alive (Table 1). Median follow-up of the patients alive at last follow up evaluation was 30.4 months (range 2.5 to 81.2 months). Three patient-specific variables were associated with OS in all models: age before RT, disease stage, and receipt of chemotherapy. Prescription dose, mean lung dose, lung V20, radiation technique (3D-CRT vs IMRT), timing of chemotherapy (concurrent vs sequential), tumor laterality, tumor site and volume were not significant.

### 3.1. Patient Specific Covariates

Overall Survival was worse for older patients (HR = 1.04 per year, P = 0.01), worse for stage IIIB vs IIIA (HR = 1.78, P = 0.02), and better for patients who received chemotherapy (HR = 0.46, P = 0.04).

### 3.2. Whole Heart Dosimetry Using KC-Lasso Multivariate Analysis

KC-lasso identified a dose threshold, D*, above which the dose volume variables, *V*_*D*≥D*_ become predictive for OS. In the dense fit, D* = 51 Gy, *i.e.*, all the coefficients corresponding to dosimetric variables with *D* < 51 Gy are zero while the first non-zero coefficient occurs for V_51_ and all the coefficients with *D* ≥ 51 Gy are non-zero. In the sparse fit, D* = 55 Gy. Both results are summarized in Table 2.

### 3.3. Whole Heart Dosimetry Using Single *V_D_* Index

Table 3 shows p-values associated with *V_D_* indices obtained by fitting a family of Cox models, each using a single *V_D_* index to represent heart dosimetry, spaced in 5 Gy increments from V5 to V60. Heart V55 predicted OS in a statistically significant manner, while V50 and V60 were nearly statistically significant. The hazard ratios (HRs) for OS are worse for increasing heart V55 (HR 1.044 per 1% increase in heart volume exposed to at least 55 Gy, P = 0.03) (Table 4).

### 3.4. KC-Lasso Consistency Check

The two variants of KC-Lasso (Table 2) and a conventional model (Table 4) provide a consistency check on the estimates of Hazard Ratio (HR) with both approaches. The ratio between coefficients in “dense” and “sparse” fit of KC-Lasso (Table 2) is approximately 1:5, which is the same as the step size ratio, hence both models will evaluate to a similar HR value. Similarly, the value of the coefficient in a conventional model is 0.043 (Table 4 and Table 1S), which is comparable to coefficients in the “sparse” KC-lasso fit (Table 2), and will thus yield a similar estimate of HR. All models are approximations and one does not expect an exact agreement among them, but one does expect a reasonable consistency of HR estimates.

### 3.5. Alternative Statistical Approaches

The three alternative statistical approaches (Lasso and Fused Lasso without knowledge constraints and Elastic Net without knowledge constraints) generated fits to data which were of comparable statistical significance to KC-Lasso but each of the three approaches had features which were difficult to interpret, like negative correlation coefficients or isolated correlation coefficients at a single dose. Hence knowledge based constraints, similar to constraints in KC-Lasso, are likely needed to create models which are both intuitively understandable and more likely to be generalizable to other data sets. A detailed discussion of the comparisons among competing statistical techniques can be found in the supplemental section (Supplement S2.4).

## 4. Discussion

We used advanced statistical techniques to overcome limitations of conventional statistical methods which are often used to search for associations between heart dosimetry and OS in lung cancer patients. Conventional analyses use the Cox model with preselected DVH variables (in a univariate or multivariate setting) and seek to establish statistically significant associations between DVH variables and OS. These approaches raise False Discovery (FDR) concerns and ignore strong correlations between DVH variables, which can lead to variable results when studies are compared (Table 5) [11]. Additional discussion of the limitations of the univariate approach is provided in the supplemental section (Supplement S2.3).

The model introduced in our work (KC-Lasso) treats the entire DVH as input and finds a contiguous range of DVH variables predictive for OS (the sensitivity range). However, the model itself cannot be used directly in commercially available optimization packages which set thresholds on individual indices only. Hence, we supplemented our analysis with a conventional approach, which used a single DVH variable to represent heart dosimetry in a multivariate Cox model and searched for the model in which this variable had the greatest statistical significance (Table 3 and Table 4). We argue that the variable with greatest statistical significance can be selected without concerns for FDR, as long as it belongs to the “sensitivity range” selected by the KC-Lasso model. Clinically, the conventional approach would be used to establish optimization constraints, by setting a limit on the Hazard Ratio, while the more complete KC-Lasso model could be used to evaluate the Hazard Ratio in the treatment plan. A more detailed discussion of the limitations of the conventional model can be found in the supplemental section (Supplement S2.3).

Our findings build upon prior studies that also show an association between high doses to relatively small volumes of the heart and decreased survival in NSCLC [7] [8] [10] [17–24]. Consistent with the findings of other investigators, our model predicts worse OS for older age [25], more advanced stage [26], and lack of chemotherapy [27].

Hazard ratios for OS associated with heart irradiation in our study are of comparable magnitude to the HRs for older age. Using the conventional model approximation as an illustration, each additional 1% of heart receiving ≥55 Gy carries similar OS impact to an additional year of older age (Table 4).

Table 5 provides a summary of the existing literature associating heart dose with OS [4] [7] [8] [10] [17–24] [28] [29] [30] [31] [32]. While our study found a range of doses for which DVH variables were associated with OS (V51 - V60), other studies have identified V30 [7] [17], V40 [8] [18], V50 [10] [19] [22], V55 [19], maximum heart dose [18], or mean heart dose [32]. The variability among studies may be attributable to strong correlations between DVH parameters which are caused by the physical properties of radiation beams (Supplement S2.3). The strength and pattern of such correlations may depend on treatment delivery techniques, which change over time and may thus affect each study differently. Advanced statistical techniques, such as the techniques employed in our study, confer an advantage of systematically examining the entire DVH, while accounting for correlations between DVH variables. Results in Table 2 show that all *V_D_* indices in the “sensitivity range” contribute to the Hazard Ratio. Additional discussion of potential reasons for discrepancies among studies is provided in the supplemental section (Supplement S2.3).

The etiology bridging the gap between heart dose and survival has yet to be confirmed. A recent systematic review details the existing literature on dosimetry and cardiac endpoints across pediatric, breast, lung, esophageal, and hematologic malignancies, emphasizing risk for coronary disease, valvular disease, arrhythmia, and pericardial disease after thoracic RT [24]. The apparent importance of upper heart substructures and specifically the superior right heart [9] [20] [30] [33] suggest radiation damage to the cardiac conduction system may impact survival in NSCLC patients. Several recent findings support this hypothesis. Among NSCLC patients treated on prospective dose-escalation trials at University of North Carolina, 11% had documented arrhythmia at 26 months after RT [3]. However, if radiation caused transient fatal arrhythmias, they would not likely be identified and may simply be recorded as deaths due to lung cancer. At 6 months after thoracic RT for locally advanced NSCLC, Vivekanandan *et al*. [9] found ECG changes in 38% of patients and ECG changes were associated with worse OS on multivariate analysis. Adding to this evidence, we have recently published [33] an expanded analysis of 3-dimensional dose distributions in the heart, for the same patient cohort as the present study, which found that the dose to the right-superior portion of the heart was most responsible for the decreased OS. More detailed cardiac evaluation of NSCLC patients receiving thoracic RT is necessary to evaluate this hypothesis.

We acknowledge the limitations of our study. Although our findings were statistically significant, our sample size is limited. Of the 16 studies in the past 10 years that have found an association between heart dose and survival, 12 included more patients than the present study (Table 5) [4] [7] [8] [10] [17–23] [28] [29] [30] [31]. Moreover, our data only included the clinical outcome of OS without any other clinical outcomes like cardiac events. Multivariate analysis mitigates the possibility of confounding by other disease and treatment-related variables but cannot exclude confounding by unaccounted for variables. Some studies have suggested confounding by or interactions with immunosuppression [22], pre-existing coronary heart disease [32], lung dose [34], or extent of mediastinal lymph node involvement [35]. Because of limited sample size we only performed Leave One Out Cross Validation and were not able to perform sample subdivision into model fitting and validation parts. Additional validation must be left to future work with an expanded data sample.

Based on our findings and the existing literature, high dose to the heart should be avoided whenever possible. For patients with NSCLC treated with conventionally fractionated RT, heart doses ≥51 Gy may decrease OS. The superior right heart may be the most at-risk for radiation induced toxicity. Data shown in Table 2 and Table 4 (heart DVH, age, stage, receipt of chemotherapy) can be used to calculate an individualized HR for OS for every stage III NSCLC patient undergoing thoracic RT (Supplement S1).

## 5. Conclusion

Among stage III NSCLC patients undergoing thoracic RT, worse OS is associated with higher heart dose, older age, more advanced stage, and lack of chemotherapy. Doses higher than 51 Gy were predictive for reduced OS, while heart V55 appeared to provide the best estimate of OS for setting treatment planning constraints, with HR 1.044 per 1% increase of heart volume exposed to at least 55 Gy.

## Supplementary Material

1

## Figures and Tables

**Table 1.** Baseline patient and treatment characteristics. Treatments were conventionally fractionated at 1.8 Gy - 2.0 Gy per fraction.

	N (%)	Median (Range)
Follow-Up of the Surviving Patients (months)		30.4 (2.5 - 81.2)
Follow-Up for All Patients (months)		18 (1.2 - 81.2)
Age (years)		70.5 (41.7 - 91.1)
Prescription Dose (Gy)		62 (43.1 - 74.0)
		1.8
Mean Lung Dose (Gy)		13.6 (4.4 - 22.4)
Lung V20 (%)		23.9 (5.7 - 41.5)
Tumor Volume [cc] (CTV)		118.5 (1.1 - 706)
Technique 3D-CRT	49 (41.2%)	
IMRT	70 (58.8%)	
Stage IIIA	72 (60.5%)	
IIIB	47 (39.5%)	
Chemo Yes	106 (89.1%)	
No	13 (10.9%)	
Laterality Left	44 (37%)	
Right	74 (62.2%)	
Undefined	1 (0.8%)	
Site Lower Lobe	26 (21.8%)	
Middle Lobe	5 (4.2%)	
Upper Lobe	80 (67.2%)	
Bronchus	5 (4.2%)	
Undefined	3 (2.5%)	
Alive at Last Follow-up	47 (39.5%)	
Deceased at Last Follow-Up	72 (60.5%)	

**Table 2.** Summary of coefficients for *V_D_* features which are predictive for OS in the KC-Lasso model. Results are shown for two models, each using an array of *V_D_* indices as input. In the “dense” model indices are spaced by 1 Gy, whereas in the “sparse” model indices are spaced by 5 Gy. For both models, coefficients are zero in the V1 - V50 range, and increase with dose thereafter. The mathematical formula needed to calculate the hazard ratio is shown at the bottom of the table. The p-value associated with dosimetric variables is shown in the last column. Note that weights in the “dense” model are approximately 5 times lower than the weights in the “sparse” model, which means that weights scale in proportion to the dose step. Both models will evaluate to a similar Hazard Ratio, showing the consistency between the two models. Summary of KC-Lasso DVH feature.

DVH index	V1 - V50	V51	V52	V53	V54	V55	V56	V57	V58	V59	V60	p-value
*β_dense_*	0.0	0.003	0.0031	0.0033	0.0035	0.0038	0.004	0.0046	0.005	0.0055	0.006	0.019
*β_sparse_*	0.0	--	--	--	--	0.021	--	--	--	--	0.033	0.02
KC-Lasso model hazard ratio = $e^{\underset{i}{\sum }{\beta_{i}}^{*}\left(\left(V\_D_{i}\right)\left[\%\right]\right)}$												

**Table 3.** A summary of P-values associated with DVH indices for a family of Cox models that represent heart dosimetry as a single, whole heart DVH. Index *V_D_* indicates percentage of heart volume receiving a dose greater or equal to D[Gy]. P-values are lowest in the same range of *V_D_* as non-zero indices of the KC-Lasso model. Since the lowest p-value is associated with V55, which is also located in the middle of the KC-Lasso range, we choose V55 as the best approximation which can be used in treatment planning. The Hazard Ratio associated with V55 is HR = 1.044 for each 1% of the heart volume exposed to at least 55 Gy.

DVH index	V5	V10	V15	V20	V25	V30
P-value	0.55	0.53	0.46	0.37	0.31	0.42
DVH index	V35	V40	V45	V50	V55	V60
P-value	1.00	0.65	0.33	0.09	0.03	0.08

**Table 4.** Multivariate Cox model for OS using whole heart V55 as a single DVH index representing heart dosimetry. V55 represents percentage of whole heart volume receiving dose 55 Gy, or greater. The Hazard Ratio for cardiac toxicity can be calculated as *HR_cardiac_* = *e*^0.043*V55^ ≅ (1.044)^*V*55^. Equations and examples needed to calculate an individualized HR for OS using a specific patient’s variables are provided in the Supplement.

Model	Heart V55	
	HR	P-value
*V_D_* (per 1%)	1.044	0.03
Age (per year)	1.04	0.01
Stage IIIB	1.78	0.02
Chemotherapy (Concurrent or Sequential)	0.46	0.04

**Table 5.** Studies published since 2008 reporting an association between survival outcomes and heart dose.

Study	N	Follow-up	Type of Cancer	RT Technique (s)	RT Prescription	Major Finding (s)
Odense Univ.^r^ Schytte, 2010	250	7.9 yrs^^^	NSCLC	3D-CRT	60 - 80 Gy	LV mean ≥ 14.5 Gy → ↓OS (p = 0.06)
Euro2K^r^ Tukenova, 2010	4122	26 yrs^^^	Pediatric	2D-RT	NR	Heart mean 1 Gy → ↑cardiac death excess RR 60% Heart mean 1-4.9 → ↑cardiac death RR 2.5 Heart mean 5-14.9 Gy → ↑cardiac death RR 12.5 Heart mean ≥15 Gy → ↑cardiac death RR 25.1
RTOG 0617^a^ Bradley, 2015	544	1.9 yrs^^^	NSCLC	3D-CRT (51.5%) IMRT (48.5%)	60 Gy (57.5%) 74 Gy (42.5%)	Heart V5 → ↓OS HR 1.007 per 1% Heart V30 → ↓OS
RTOG 0617^a^ Eaton, 2016	495	NR	NSCLC	3D-CRT (51.7%) IMRT (48.3%)	60 Gy (58.2%) 74 Gy (41.8%)	Heart V50 → ↑Grade 5 AE
RTOG 0617^a^ Chun, 2017	482	1.8 yrs^^^	NSCLC	3D-CRT (53%) IMRT (47%)	60 Gy (58%) 74 Gy (42%)	Heart V40 → ↓OS HR 1.01 per 1%
William Beaumont^r^ Johnson, 2017	178	1.4 yrs^^^	NSCLC	3D-CRT (38.4%) IMRT (61.6%)	64 Gy^^^	Heart V30 → ↓OS HR 1.013 per 1%
Univ. of Manchester^r^ McWilliam, 2017	1101	3 - 36 mos	NSCLC	3D-CRT IMRT SBRT (7.4%)	55 Gy (non-SBRT) 60 Gy in 5 fx (SBRT)	Heart base mean > 16.3 Gy → ↓OS HR 1.25 (non-SBRT) Heart base mean > 8.5 Gy vs < 8.5 Gy → ↓OS (non-SBRT) Heart base mean > 6.3 Gy → ↓OS HR 2.11 (SBRT)
Mayo Clinic PCI^r^ Sio, 2017	76	5.5 yrs^^^	Thoracic -Breast (57%) -NSCLC (17%) -Upper GI (11%)	2D-RT 3D-CRT IMRT (1.3%) SBRT (2.6%)	53.4 Gy*	Heart mean → ↓OS HR 2.01 per 1 Gy Heart mean → ↑non-cancer death HR 1.49 per 1 Gy Heart max → ↓OS after PCI HR 1.02 per 1 Gy Heart V40 → ↑non-cancer death HR 1.32 per 1%
Washington Univ.^r^ Speirs, 2017	322	1.2 yrs^^^	NSCLC	3D-CRT (60%) IMRT (40%)	66 Gy^^^	Heart V50 → ↓OS HR 1.23 per 1% Heart V55 → ↓OS HR 1.85 per 1% (CRT only)
Multicenter^r^ Stam, 2017	803	2.9 yrs^^^	NSCLC	SBRT	54 Gy in 3 fx^^^	LA max → ↑non-cancer death HR 1.005 per 1 Gy SVC D90 → ↑non-cancer death HR 1.025 per 1 Gy
Meta-Analysis^a^ Taylor, 2017	40,781	10 yrs^^^	Breast	2D-RT	NR	Heart mean → ↑cardiac death RR 1.04 per 1 Gy
IDEAL-CRT^a^ Vivekanandan, 2017	78	35 mos^^^	NSCLC	3D-CRT (“most”) IMRT (“some”)	67.7 Gy*	LA wall V63 > 2.2% → ↑OS HR 1.52
Washington Univ.^r^ Contreras, 2018	400	17 mos^^^	NSCLC	3D-CRT (59%) IMRT (41%)	66 Gy^^^	Heart V50 → ↓OS HR 1.02 per 1%
Princess Margaret^r^ Wong, 2018	189	35.3 mos^^^	NSCLC	SBRT	48 Gy in 4 fx (47.1%) 54 - 60 Gy in 3 fx (24.3%)	Ventricle max → ↑non-cancer death HR 1.02 per 1 Gy
Multicenter^a^ Xue, 2019	94	58 mos^^^	NSCLC	3D-CRT	70 Gy^^^	Pericardium V30 > 29% → ↓OS (HR 1.019 per 1%) Pericardium V55 > 21% → ↓OS (HR 1.03 per 1%)
Dana-Farber Atkins 2019	748	20.4 mos^^^	NSCLC	3D-CRT (78.1%) IMRT (21.9%)	66 Gy^^^	Heart mean → ↑ACM HR 1.02 per 1 Gy With pre-existing CHD, heart mean ≥ 10 Gy → ↑ACM HR 1.34 Without pre-existing CHD, no association between heart mean and ACM
**Present Study** ^r^ Niska 2019	119	1.5 yrs^^^	NSCLC	3D-CRT (41.2%) IMRT (58.8%)	62 Gy^^^	Heart V55 → ↓OS HR 1.044 per 1% Heart V51 – V60 range predictive for OS

Abbreviations: 2D-RT, 2-dimensional RT; 3D-CRT, 3-dimensional conformal RT; ACM, all-cause mortality; AE, adverse event; CHD, coronary heart disease; D(xx), minimum dose to xx% of the volume; fx, fractions; GI, gastrointestinal; Gy, Gray; HR, hazard ratio; IMRT, intensity-modulate RT; LA, left atrium; LV, left ventricle; mos, months; NR, not reported; NSCLC, non-small cell lung cancer; OS, overall survival; PCI, percutaneous coronary intervention; RR, relative risk; RT, radiotherapy; RTOG, Radiation Therapy Oncology Group; SBRT, stereotactic body RT; SVC, superior vena cava; univ., university; V(x), volume receiving at least x Gy; yrs, years.

^ indicates median

* indicates mean

a indicates prospective

r indicates retrospective.
