# Supplementary material for: Using Novel Statistical Techniques to Accurately Determine the Predictive Dose Range in a Study of Overall Survival after Definitive Radiotherapy for Stage III Non-Small Cell Lung Cancer in Association with Heart Dose
Source: J Cancer Ther. Author manuscript; Available in PMC 2021 Nov 18. (PMC8601657; doi:10.4236/jct.2021.129044)
Supplement: 1 [file NIHMS1744438-supplement-1.pdf]

Supplemental

S1. Clinical Applications Supplement

The Cox proportional hazards regression model can be written as follows:

$$h(t) = h_0(t)\exp(b_1 X_1 + b_2 X_2 + \cdots + b_n X_n)$$

where $b_i X_i$ represent potential predictors and $h_0(t)$ a baseline risk when all predictors are set to zero.

S1.1. Whole Heart DVH Analysis

Table S1 shows fit coefficients, hazard ratios, and p-values which were associated with significant predictors of overall survival in the model which included V55Gy for the whole heart DVH.

S1.2. Example Applications Using Whole Heart V55Gy

1) With the "reference patient" defined as stage IIIA, no chemotherapy, 70.5 years old, and heart V55Gy = 0%, the individualized hazard ratio for an actual patient with stage IIIB disease, who received chemotherapy, and whose heart V55Gy was N%, can be computed as (from Table 4):

$$\text{HR} = 1.78 \times 0.46 \times 1.04^{(\text{age}-70.5)} \times 1.044^{N}$$

The interpretation of HR = X is that the likelihood of dying per unit of time for the actual patient is X-fold greater than the likelihood of dying per unit of time for the reference patient.

2) Holding the other variables constant, for a patient whose V55Gy to the whole heart is N%, the associated hazard ratio for cardiac radiation exposure can be calculated as

$$\mathrm{e}^{N \times 0.043} \text{ or } 1.044^{N}.$$

Consider two patients who are the same age, have the same cancer stage, and both received chemotherapy, but differ only by extent of heart irradiation. Patient A received heart V55Gy = 0%. Patient B received heart V55Gy = 10%. Using the coefficient from the second column of Table S1, the individualized hazard ratio for patient A is

$\mathrm{e}^{0 \times 0.043} = 1.0$. The individualized hazard ratio for patient B is $\mathrm{e}^{10 \times 0.043} = 1.537$. The likelihood of dying per unit of time is 1.537 times higher for patient B than for patient A.

Table S1. Model coefficients and associated hazard ratios in the model with whole heart DVH.

Predictor X_i	Coefficient b_i	Hazard Ratio	p-value
Stage IIIB	0.57	1.78	0.016
Chemotherapy Use	−0.79	0.46	0.039
Age before RT	0.04	1.04/1 year	0.012
V55Gy	0.0430	1.044/1% of volume	0.034

Another way to obtain the estimate of hazard ratio associated with cardiac irradiation is to use the hazard ratio listed in Table 4. Using the same example patients as before, the hazard ratio for patient B is $1.044^{10} = 1.538$.

3) If two groups of patients have hazard ratios equal to HR_1 and HR_2, the ratio of the mean survival times between the two groups can be approximated by $\frac{HR_1}{HR_2}$.

S2. Statistical Analysis Supplement

S2.1. Radiobiological and Clinical Motivation for "Knowledge Constraints" in KC-Lasso

1) Introduction

Dose-volume analysis is one of the primary tools used in phenomenological modelling of clinical toxicity in radiation therapy. Dose volume analysis reflects the basic clinical and radiobiological insight that the likelihood of clinical toxicity depends on both the dose level and the volume to which the dose is applied. In general, larger doses and larger volumes to which dose is applied lead to greater likelihood of toxicity. The dominant effect of depositing dose in a volume of tissue is cell kill (cell depletion), usually described as cell Survival Fraction (SF), which is the proportion of cells that survive an irradiation. The SF is related to dose by a Linear Quadratic model equation $\text{SF} = e^{-\alpha * \text{BED}}$ where BED is Biologically Equivalent Dose (BED) related to the physical dose through a linear quadratic equation $\text{BED} = D\left(1 + \frac{d}{\alpha/\beta}\right)$. In the discussion that follows we will equate the physical dose D with BED and refer to both as "dose".

Irradiating a volume of tissue with a dose level "D" can lead to one of the three categories of outcomes:

a) The SF may be high enough that the tissue can compensate for lost cells and there is no clinical toxicity observed.

b) The SF is low enough that the tissue may not be able to fully compensate for lost cells which can lead to transient or permanent toxicity. The toxicity is transient if the tissue can rebuild itself over time and permanent if the tissue can no longer rebuild itself. In this regime, the onset of toxicity is probabilistic and may additionally depend on patient specific characteristics, like age, state of health or genetics.

c) The SF is so low that toxicity will inevitably occur, for all patients.

The (1)-(3) states are typically modelled by the sigmoid (logistic) curve. The Region (1) corresponds to the beginning part of the curve, Region (2) corresponds to the rising part of the curve, and Region (3) corresponds to the saturation region of the logistic curve.

2) The Linear Predictor in KC-Lasso

The linear predictor in KC-Lasso assumes the following form

$$\eta = \beta_0 + \beta_1 V_{D_1} + \cdots + \beta_p V_{D_p}$$

where V_{D_i} is the percentage of organ volume with dose D_i, or greater.

3) Positivity Condition in KC-Lasso

The positivity condition in KC-Lasso says that all coefficients β_i have to be greater or equal to zero. ($\beta \geq 0$)

The motivation for the positivity condition is that any dose, applied to any volume, kills cells (SF < 1). Such irradiation can produce no risk (Region (1)), some risk (Regions (2)-(3)), but it cannot reduce risk, which would be implied by negative coefficients.

4) Monotonicity Condition in KC-Lasso

Consider one of the elements in the linear predictor:

$$\beta_i V_{D_i}$$

Let us fix the value of V_{D_i} at an arbitrarily chosen value of 0.05 (5% of the volume irradiated to the dose D_i or higher). What should the contribution of this term to risk be when D_i increases? An increase in dose means that the surviving fraction (SF) decreases. A decrease in SF means that the contribution of this term to risk has to increase (Region (2), increasing risk) or stay the same (Region (3), saturated risk).

Returning to the full linear predictor feature, one thus observes that, for a fixed value of V_{D_i} (e.g. 0.05) the contribution of consecutive terms to the feature has to increase, or stay the same, as the dose increases. This argument leads us to the monotonicity condition, which says that consecutive coefficients must be greater or the same as their predecessors:

For any two dose levels d_1, d_2, with $d_1 \leq d_2$, let β_{d_1} and β_{d_2} be the coefficients for V_{d_1} and V_{d_2}, respectively. Then, $\beta_{d_1} \leq \beta_{d_2}$.

S2.2. Internal Validation

Since the KC lasso method is based on fused lasso, there are two tuning parameters we need to determine, *i.e.*, the L1 penalty for the coefficients and the L1 penalty for the steps of the coefficients. We use the work by Dai and Breheny [15] who concluded that a method of leave-one-out cross validation with linear predictors works the best for a Cox model. To be more specific, suppose that there are n patients in the data. In the *i*-th iteration, we leave the *i*-th patient out and use the remaining *n*-1 patients to fit the Cox model with KC lasso. Then the 'linear predictor' of the *i*-th patient can be defined as $\hat{\eta}_i = \vec{X}_i^{\mathrm{T}} \hat{\beta}^{-i}$, where $\vec{X}_i^{\mathrm{T}}$ is the feature vector for *i*-th patient and $\hat{\beta}^{-i}$ is the estimated coefficient vector of the model without *i*-th patient. After we get "linear predictors" for all patients, we can define the predictive accuracy based on the "linear predictors" by

$$L(\hat{\eta}) = \prod_{i=1}^{n} \left[\mathrm{e}^{\hat{\eta}_i} \Big/ \sum_{j \in R(t_i)} \mathrm{e}^{\hat{\eta}_j} \right],$$

where t_i is the event time or last follow up time of *i*-th patient. $R(t_i)$ is the set of patients at risk at time t_i. The cross validation error for linear predictors is then defined by $\mathrm{CVE} = -2\log\left(L(\hat{\eta})\right)$. We find the set of tuning parameters with the lowest CVE (cross validation error).

S2.3. Limitations of the Univariate Analysis

In **Figure S1**, we show p-values associated with the dosimetric variable V_D ina Cox model which contains patient specific covariates (age, chemotherapy and stage) and one V_D index at a time. We effectively scanned the V_D space and recorded the p-value associated with each V_D. One observes a monotonic decline of p-values, starting between doses of 35 Gy - 40 Gy and continuing towards the minimum near V_{55Gy}. The monotonic decline in such a broad range of doses is caused primarily by correlations among indices for this population of patients, convolved with the threshold dose. The magnitude of correlations in the present data is shown in **Figure S2**. Correlations among indices are determined by the combination of three factors: 1) anatomic variability among patients, 2) variability in tumor location and volume, 3) dose distributions which are characteristic for the delivery methods being used. The magnitude of p-value at the minimum also depends on the sample size. If one sets an arbitrary threshold of p-value at p = 0.05, this threshold will be crossed at a dose level which depends both on the sample size and on the pattern of correlations, which can vary in different studies due to differences in treatment delivery methods being used. Hence, the method of searching for the dose threshold with univariate analysis tends to produce threshold doses which are too low and can vary among studies. A more advanced statistical technique, which explicitly "corrects" for correlations, needs to be used to detect a threshold dose which will remain consistent for all studies and is generalizable to future clinical applications.

If one considers multiple V_D indices as uncorrelated, independent variables, p-value obtained for each of these variables should be subjected to multiple comparisons correction. The correction will depend on the size of the scanning

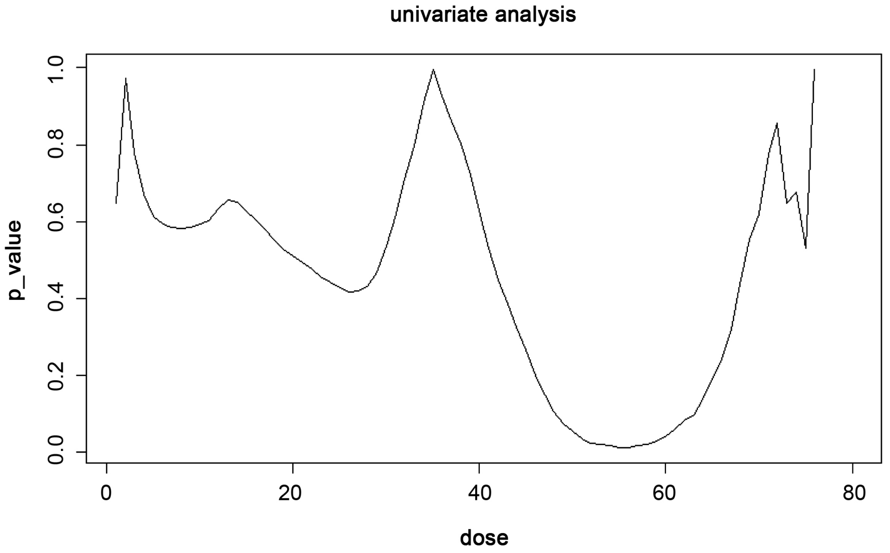

Figure S1. P-values associated with V_D covariate in a Cox model with patient specific variables (age, stage and chemotherapy) and one dosimetric covariate at a time. Doses are in Gy and a 1 Gy step was used in the analysis. Data in **Table 3** were sampled from this figure.

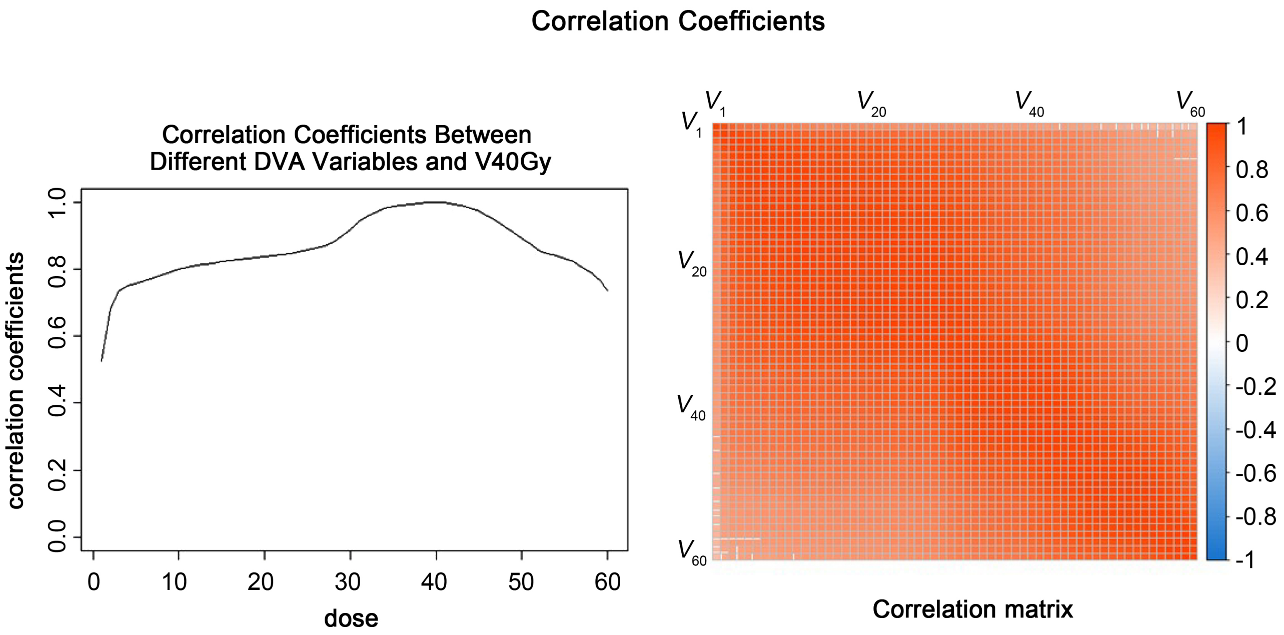

Figure S2. Correlations among V_D indices, the correlation matrix on the right side and a single section through the matrix at the level of V_{40} on the left side.

step and may prevent the p-values from reaching the threshold of statistical significance for patient cohorts of a realistic size. Adjusting the step size to reach the threshold of statistical significance is not well justified and reduces the precision of searching for a dose threshold which is associated with the clinical outcome. The magnitude of this problem is illustrated in **Figure S3**, using the V_D step size of 1 Gy. Red bars show p-values obtained without multiple comparison correction, while blue bars show p-values adjusted for the correction. The threshold of statistical significance would not have been reached in the present patient cohort if p-values were corrected for multiple comparisons.

S2.4. Alternative Statistical Methods

KC-Lasso has been designed for the present study to address the problem of correlations among dosimetric variables. One can reasonably ask whether other statistical techniques could perform equally as well as KC-Lasso model. To address this question we compared KC-Lasso (Knowledge Constrained, Fused Lasso) to Elastic Net [16], Lasso [13] and Fused Lasso [14] models. We make a comparison by first deriving the coefficients for the V_D variables in each model which creates a linear predictor (feature) for each. We then compare the p-value associated with the feature in an unpenalized Cox model containing patient specific covariates. All four models were associated with very similar p-values, as summarized in **Table S2**.

All four models produced very similar survival curves for surviving patients. KC-Lasso showed some differences in survival curves for deceased patients. Two examples are shown in **Figure S4** and **Figure S5**.

Table S2. P-values associated with the linear predictor in four models in an unpenalized cox model with patient specific covariates.

Model	p-value
KC-Lasso	0.0122
Lasso	0.0134
Fused Lasso	0.0123
Elastic Net	0.0122

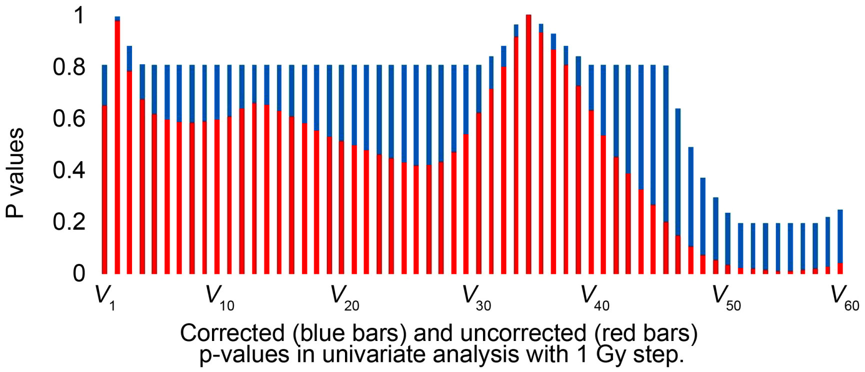

Figure S3. An illustration of the effect that multiple comparisons correction would have on p-values in the univariate analysis for the present study.

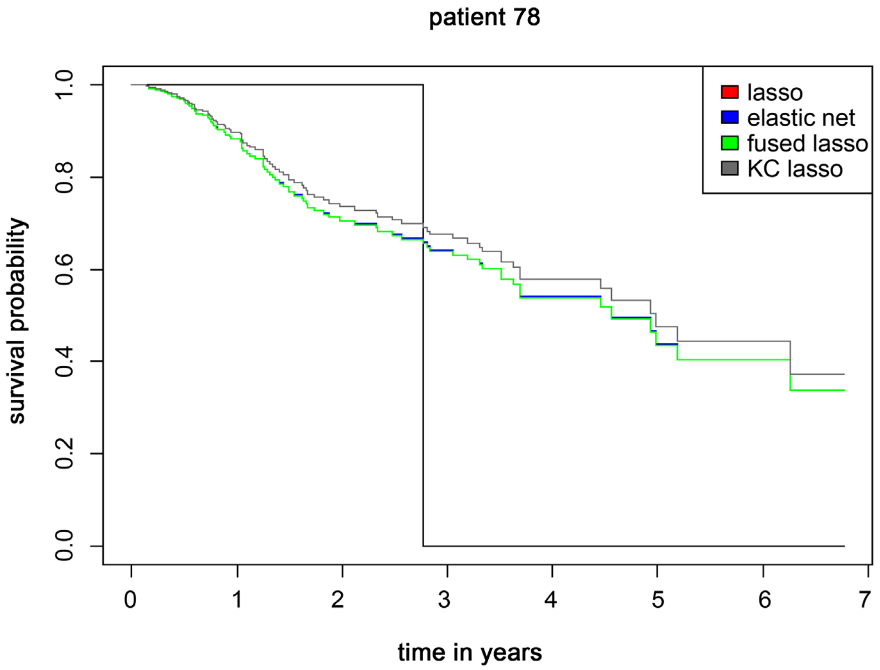

Figure S4. An example of survival curves for a surviving patient.

All four models perform similarly on the same data set, though one could argue that KC-Lasso is showing slightly better prediction of survival probability. The primary difference between the models is in the choice of coefficients for the linear predictors that each model makes. The coefficients chosen by the Elastic

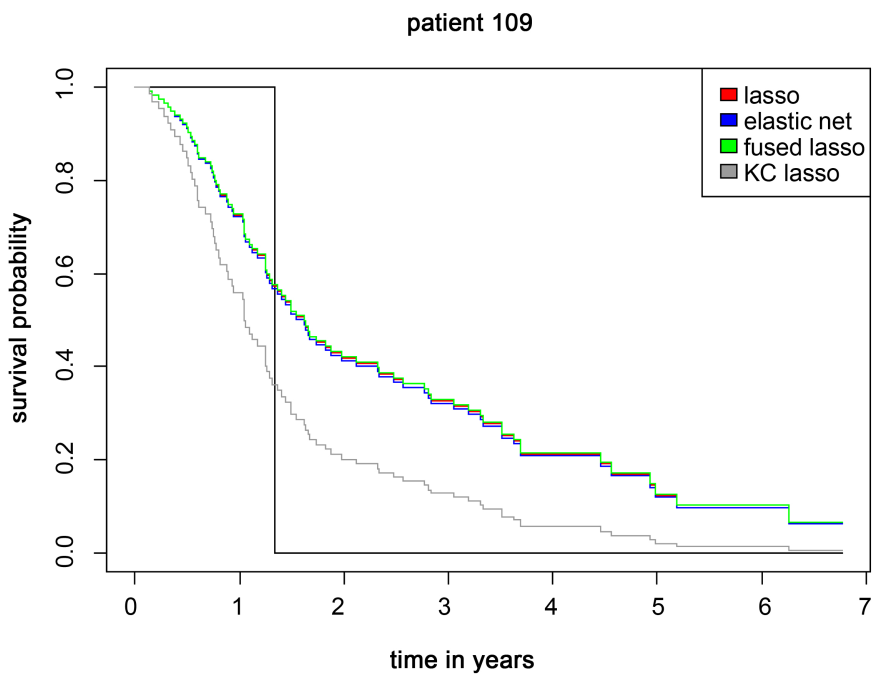

Figure S5. An example of survival curves for a deceased patient.

Net (**Figure S6**), Lasso (**Figure S7**) and Fused Lasso (**Figure S8**) models are shown below.

One observes that both the Elastic Net, Lasso and Fused Lasso models selected negative coefficients. Negative coefficients are biologically implausible because they imply a possibility that the irradiation of tissue at risk improves the odds of survival. A second biologically implausible feature is the selection of a single V_D index (or a small group of indices) without a simultaneous selection of indices at higher doses. Higher doses are always associated with lower cell survival fraction in the volume. Radiobiology suggests that lower cell survival fraction in a fixed volume of tissue should always be associated with higher likelihood of clinical complications, or at least the same likelihood of complications if cell survival fractions are so low that the adverse clinical outcome is virtually assured. Consequently, once the first V_{Dthr} (threshold dose) is selected, all $V_{D>Dthr}$ should also be selected and their coefficients should be higher (more risk) or equal (saturated risk). The constraint on the maximum dose in the analysis must be imposed by the maximum dose available in the data.

In summary, KC-Lasso is not the only statistical model that can be successfully fit to the present data set. However, KC-Lasso has been designed to satisfy "common sense" boundary conditions (positivity and monotonicity conditions imposed on coefficients) as well as to account for correlations between V_D indices. The purpose of this design has been to make the results of the model easier to interpret intuitively and to be more generalizable.

S2.5. Tumor Volume as a Patient Specific Covariate

Tumor volume can influence the likelihood of OS and can also influence

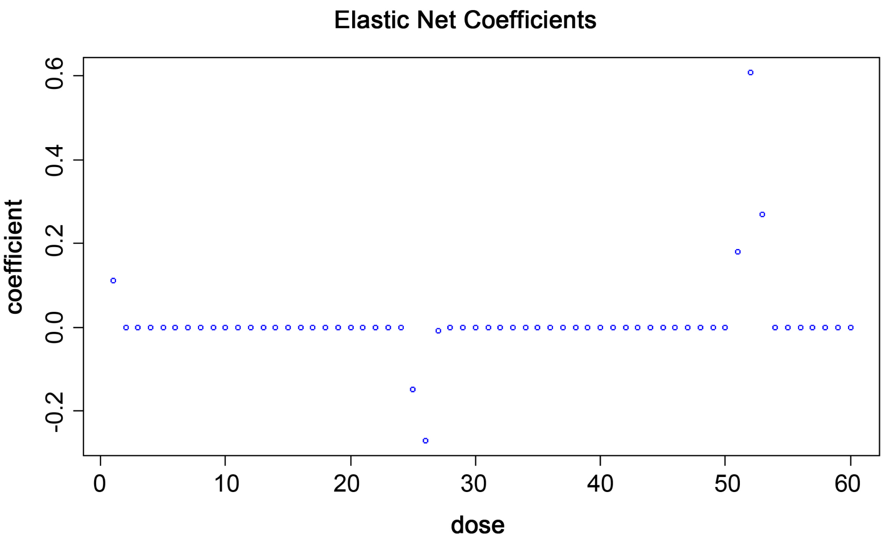

Figure S6. Coefficients selected by the elastic net model.

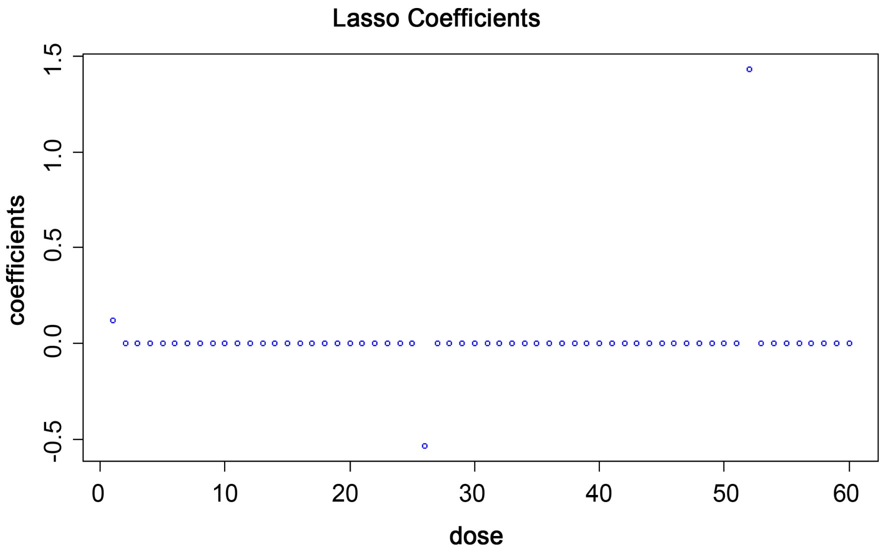

Figure S7. Coefficients selected by the lasso model.

the irradiation of the heart. Tumor volume was difficult to assess in this retrospective study because physicians frequently broke the target hierarchy (GTV-CTV-PTV-ITV) during contouring and thus forced estimates of the tumor volume with assumptions. We estimated the tumor volume (under assumptions) but decided to substitute tumor volume with a patient specific covariate which is strongly correlated with volume, namely clinical stage (3A and 3B). The clinical stage has been reliably recorded prior to treatment and reflects added clinical risk associated with tumor progression. The correlation between our estimated tumor volume and the clinical stage is summarized in **Table S3**.

When volume alone is used in the Cox model it is not a predictor for the OS ($p = 0.35$). When volume alone is used in the Cox model with chemotherapy and age it is predictive for OS with $p = 0.048$. When stage is used instead of volume,

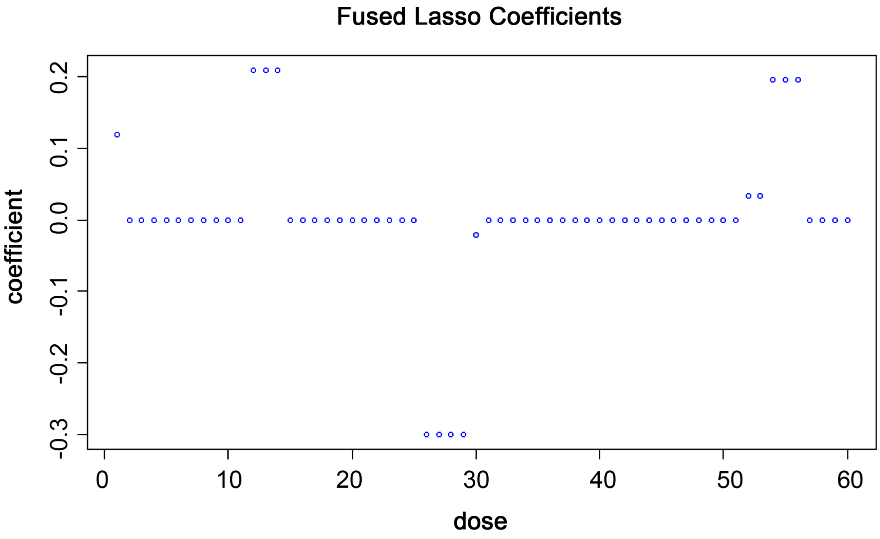

Figure S8. Coefficients selected by the fused lasso model.

Table S3. Summary of correlations between the disease stage and the estimated CTV volume.

	Estimated CTV volume (cubic centimeters)			
Stage	Mean	STDEV	MIN	MAX
3A	118.5	123.1	1.1	619
3B	180	143.6	4.7	706
t-test p-value	0.0083	N/A	N/A	N/A

the stage (3A/3B) is a predictor for OS with p = 0.024. When volume and stage are included simultaneously, neither one is the predictor with p-values p = 0.18 for volume and p = 0.07 for stage. Given that stage and estimated volume are strongly correlated, we chose to include stage alone in the analysis.
